# Transcriptome analysis in patients with asthma after inhaled combination therapy with long-acting β2-agonists and corticosteroids

**DOI:** 10.7150/ijms.76013

**Published:** 2022-10-03

**Authors:** Ya-Ru Liang, I-Shiang Tzeng, Po-Chun Hsieh, Chan-Yen Kuo, Shiang-Yu Huang, Mei-Chen Yang, Yao-Kuang Wu, Chou-Chin Lan

**Affiliations:** 1 Division of Respiratory Therapy, Taipei Tzu Chi Hospital, Buddhist Tzu Chi Medical Foundation, New Taipei City, Taiwan.; 2 Department of Research, Taipei Tzu Chi Hospital, Buddhist Tzu Chi Medical Foundation, New Taipei City, Taiwan.; 3 Department of Chinese Medicine, Taipei Tzu Chi Hospital, Buddhist Tzu Chi Medical Foundation.; 4 Division of Pulmonary Medicine, Taipei Tzu Chi Hospital, Buddhist Tzu Chi Medical Foundation, New Taipei City, Taiwan.; 5 School of Medicine, Tzu-Chi University, Hualien, Taiwan.

**Keywords:** asthma, inhaled corticosteroids, long-acting beta_2_-agonists, RNA transcriptome

## Abstract

**Introduction:** Asthma is one of the major public health problems that imposes a great burden on societal, financial, and healthcare around the world. Asthma poorly affects the health-related quality of life and daily activities of patients. Treatment of asthma, including inhaled corticosteroids (ICS) and long-acting beta-agonists (LABAs), mainly aims to improve the lung function and reduce symptoms and exacerbations. Current treatment regimens are symptom-based strategies, and the status of airway inflammation after treatment is yet unknown. We conducted this study to understand the comprehensive inflammation or airway remodeling status of patients after ICS-LABA treatment through RNA transcriptome analysis.

**Materials and methods:** Eight newly diagnosed asthmatic patients and two healthy subjects were recruited in this study. Asthmatic patients underwent blood tests, lung function test, and RNA transcriptome analysis before and after ICS-LABA treatment.

**Results:** In comparison with healthy subjects, pretreatment asthmatic patients had higher expression of protein tyrosine kinase and related signaling pathways. After ICS-LABA treatment, the expression of nuclear receptor transcription coactivator, N-acetyltransferase, protein tyrosine kinase, nuclear receptor, and RNA polymerase II-activating transcription factor were downregulated. However, the post-treatment asthmatic patients still had higher expression of cysteine-type endopeptidase, endodeoxyribonuclease, apolipoprotein, and unfolded protein was still upregulated than healthy subjects.

**Conclusions:** The combination of ICS/LABAs decreased airway inflammatory and remodeling pathways. However, allergen stimulation-related pathways were still upregulated in patients after ICS/LABA treatment. The combination of medication and allergen removal is a complete strategy for asthma.

## Introduction

Asthma, a major public health concern worldwide, affected an estimated 334 million people 1. Prior studies have revealed the prevalence of asthma to be approximately 15% in many countries, and the disease is often reported in developed nations 1. The global asthma prevalence is increasing, and it is estimated that there will be 100 million new cases in the next 10 years 1. Therefore, asthma imposes a huge burden on society, finance, and healthcare around the world.

Considering the airway inflammation and obstruction, asthma often leads to symptoms of wheezing, breathlessness, chest tightness, and cough 1. People with uncontrolled asthma often have low health-related quality of life, sleep disturbance, and poor daily activity. Patients with severe asthma or those with acute exacerbation may need emergency medical care, and often experience hospitalization or even death. Asthma is the 28th leading cause of loss of years in full health, and approximately 2 million people visit the emergency room each year due to asthma exacerbations 1.

Asthma treatment is guided by Global Initiative for Asthma (GINA) strategies. In brief, the GINA guidelines recommend the initiation therapy with anti-inflammatory or bronchodilation, including inhaled corticosteroids (ICS) and long-acting beta₂-agonists (LABAs) for symptom-driven use or as a regular daily medication 2. The main goal of these treatments is to reduce airway inflammation, maintain lung function, ameliorate symptoms, decrease the risk of exacerbations and asthma-related deaths 2.

A central feature of asthma is the symptoms due to underlying airway inflammation. ICS-containing therapies are the mainstay treatment option for asthma 3. However, clinical studies have shown that many asthmatic patients do not respond to ICS-containing therapy 3. In addition, asthma treatment currently employs symptom-based strategies. However, for asthma patients, good control of symptoms may not indicate good control over airway inflammation. Therefore, we conducted this study to understand the comprehensive inflammation or airway remodeling status of patients after the standard combination of ICS-LABA treatment through RNA transcriptome analysis. A comprehensive understanding of patients' mRNA expression after treatment may help us optimize both pharmacological and non-pharmacological treatments for asthma.

## Materials and methods

### Patient recruitment

Eight newly diagnosed asthmatic subjects and two healthy subjects were recruited for this study. All these patients were adults that over 18 years of age. The diagnosed of asthma was confirmed by the provocation test. These newly diagnosed asthmatic patients were without oral steroid, ICS and LABAs before inclusion. All these patients were at GINA steps 3 and received ICS+LABAs after diagnosis 2. No patients received systemic corticosteroids or inhaled long-acting muscarinic agonists during the course of the study. The exclusion criteria were as follows: 1) smokers; 2) patients suffering from other lung diseases such as lung cancer, chronic obstructive pulmonary disease, pneumoconiosis, tuberculosis, bronchiectasis, and interstitial lung disease; 3) patients with systemic diseases such as diabetes mellitus, hypertension, myocardial infarction, congestive heart failure, and pulmonary embolism; and 4) those who did not want to participate in this study.

The criteria of healthy subjects was quite strict; there was no personal history of asthma, allergic rhinitis, gastroesophageal reflux disease, diabetes mellitus, hypertension, etc. These healthy subjects did not consume any drugs. This study was conducted after obtaining informed consent from all the participants and was approved by the ethics committee of Taipei Tzu-Chi Hospital.

### Study design

Before asthma treatment, patients underwent blood tests, including RNA transcriptome analysis, Specific Allergen Test Panel (Pharmacia Diagnostics AB, Uppsala, Sweden), immunoglobulin E (IgE), whole blood cell counts (with differential counts), hemoglobin (Hb), aspartate aminotransferase (AST), aminotransferase (ALT), total bilirubin, creatinine (Cr), blood urea nitrogen (BUN), C-reactive protein (CRP), potassium (K), sodium (Na), and chest radiographs. Demographic information was collected at baseline. After 2 months of treatment, all patients underwent blood tests. Healthy subjects also underwent blood tests, as mentioned above.

### Pulmonary function test (PFT) and methacholine challenge test (MCT)

Well-trained technicians performed MCT and PFT. PFT was performed between 8 AM and 4 PM using a spirometer (Medical Graphics Corporation; St Paul, MN, USA), as per the recommendations of the American Thoracic Society, and the European Respiratory Society. MCT was performed using a nebulizer with methacholine doses, and forced expiratory volume (FEV_1_) was measured 5 min after each provocation step. Provocative doses that caused a 20% decrease in FEV_1_ were recorded as PD_20_ 4.

### Symptom score

The Chinese version of the Asthma Control Test (ACT) questionnaire was used to assess symptoms of dyspnea, wheezing, coughing, and rescue medications 5. There are five items of ACT; the score range for each item is 0 to 5, and the total score ranges from 0 to 25. A higher score indicates better asthma control.

### Library preparation and sequencing

Purified RNA was used to prepare sequencing libraries using the TruSeq Stranded mRNA Library Prep Kit (Illumina, San Diego, CA, USA).We purified mRNA from total RNA (1 µg) using oligo(dT)-coupled magnetic beads and fragmented them into small pieces at high temperature. First-strand cDNA was synthesized using reverse transcriptase and random primers. After double-stranded cDNA was generated and adenylated at the 3′-ends of the DNA fragments, adapters were ligated and purified with the AMPure XP system (Beckman Coulter, Beverly, USA). Agilent Bioanalyzer 2100 system and real-time PCR system were used to assess the quality of libraries. Eligible libraries were then sequenced on the Illumina NovaSeq 6000 platform with 150 bp paired-end reads generated by Genomics (New Taipei City, Taiwan).

### Bioinformatics analysis

We removed the sequences from adapters and low-quality bases in the raw data using the Trimmomatic (version 0.39) program 6. We used Bowtie2 (version 2.3.4.1) as filtered reads to align the reference genomes 7. The quantification of Transcript abundance was performed by RSEM (version 1.2.28) 8. EBSeq (version 1.16.0) was used to identify differentially expressed genes 9, and the R package of cluster Profiler (version 3.6.0) was used to analyze functional enrichment of Gene Ontology (GO) terms and Kyoto Encyclopedia of Genes and Genomes (KEGG) pathways among gene clusters 10.

The FASTQ files of RNA sequencing data are available at National library of Medicine (NCBI SRA: SUB120238883).

### Statistical analysis

Continuous data were expressed as mean and standard deviation, and categorical data were expressed as frequencies and percentages. A Welch's *t*-test was used to compare parameters between asthmatic and healthy subjects. Wilcoxon signed rank test was used to compare parameters of asthmatic patients before and after treatment. Statistical significance was set at *p* < 0.05. All statistical analyses were performed using SPSS (version 24.0; SPSS, Inc., Chicago, IL, USA) and R software (version 4.2.0). *A* value < 0.10 was considered statistically significant for GO and KEGG data.

## Results

### Baseline characteristics

The characteristics and PFTs of these patients are shown in Table 1. The critical sources of allergen for asthma were dust, cats, and dogs, although there were no analytic differences with healthy subjects. There were no differences in PFT parameters between asthmatic and healthy subjects.

The comparison results of laboratory data between asthma patients and healthy subjects are shown in Table 2. The eosinophil count was higher in pre-treated asthma patients than that in healthy subjects (4.7% ± 2.9% vs. 2.0% ± 0.4%, *p* = 0.0209). IgE level was trend towards be higher in pre-treated asthma patients than that in healthy subjects, but no significant difference was observed (426.6% ± 658.0 IU/mL vs. 31.5 ± 31.4 IU/mL,* p* = 0.1101). ACT scores of pre-treated asthma patients were lower than those of healthy subjects (16.2 ± 2.9 vs. 25.0 ± 0.0,* p* <0.001). We compared post-treatment asthma patients with healthy subjects and found that the eosinophil percentage was 3.9% ± 2.8%, which was trend towards to be higher than that reported in healthy subjects (*p* = 0.0802) but trend towards a decrease than the value observed in pretreatment asthma patients but without significant difference (*p* = 0.1641). IgE levels also had trend towards a decrease after treatment, but without analytical significance (*p* = 0.5703). The ACT score of post-treatment asthma patients was 20.1 ± 2.5, which was lower than that of the healthy subjects (25.0 ±0.0, *p* = 0.0003) but higher than that of pretreatment asthma patients (16.2±2.9, *p* = 0.0156).

### Analysis of RNA transcriptomes of normal subjects and asthmatic patients

There were total 26507 genes identified. A total of 147 differentially expressed genes (DEGs) were found. The criteria of significant difference was PPEE (posterior probability of equal expression) <0.05. There was an overlap in DEG between asthma baseline/control and asthma baseline/asthma treated, SLA2.

### Differences in the RNA transcriptomes of normal subjects and pre-treatment asthmatic patients

**Figure 1:**
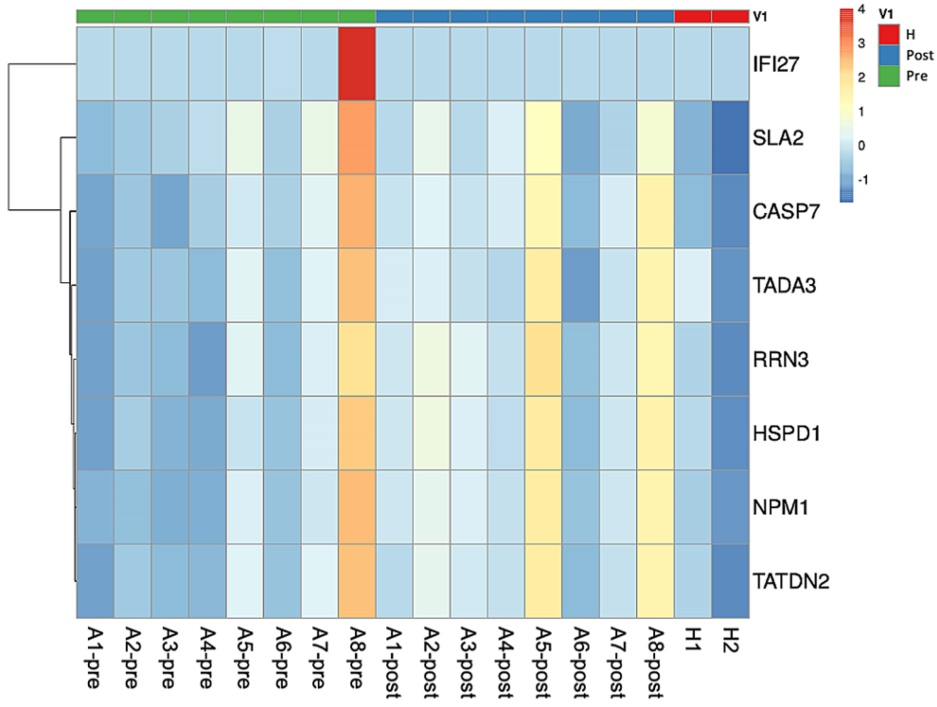
** Heatmap plot of asthmatic patients and healthy subjects.** The comparison between pre-treatment asthmatic patients and healthy subjects revealed the upregulation in *SLA2* gene expression. *TADA3*, *SLA2*, and *IFI27* genes were downregulated in asthmatic patients before and after treatment. Comparison between asthma patients after treatment and healthy subjects showed that *CASP7*, *TATDN2*, *NPM1*, *HSPD1*, and *RRN3* expression were upregulated in asthmatic patients after treatment. Abbreviations: Src-like adaptor 2: SLA2; transcriptional adaptor 3: TADA3; interferon alpha inducible protein 27: IFI27; caspase 7: CASP7; TatD DNase domain containing 2: TATDN2; nucleophosmin 1: NPM1; heat shock protein family D member 1: HSPD1; RNA polymerase I transcription factor 3: RRN3.

The comparison of the RNA transcriptomes between healthy subjects and pre-treatment asthmatic subjects revealed the upregulated protein tyrosine kinase activity, signaling adaptor activity, SRC homology 3/SRC homology 2 (SH3/SH2), which is regulated by the Src-like adaptor 2 (*SLA2*) gene, in the patient group. GO terms, regulated genes, and description of functions are shown in Table 3. The heatmap is shown in Fig. 1. For a case whose representation in the heatmap was an outlier (pre and post levels appeared to be in opposite directions compared to other patients), the case was removed for heatmap analysis.

### Changes in the RNA transcriptomes of asthma patients before and after treatment with ICS/LABAs

We compared the RNA transcriptomes of asthma patients before and after treatment with ICS/LABAs, and found downregulation in the ligand-dependent nuclear receptor transcription coactivator activity, N-acyltransferase (NAT) activity, protein tyrosine kinase activity, ligand-dependent nuclear receptor, RNA polymerase II-activating transcription factor, and SH3/SH2, which are regulated by transcriptional adaptor 3 (*TADA3*), *SLA2*, and interferon alpha-inducible protein 27 (*IFI27*) genes. The GO terms, regulated genes, and descriptions of functions are shown in Table 4 and the heatmap is shown in Fig. 1.

### Differences in the RNA transcriptomes of post-treatment asthmatic subjects and healthy subjects

The results of the comparison of the RNA transcriptomes of asthma patients after treatment with ICS/LABAs and normal subjects are shown in Table 5. The cysteine-type endopeptidase involved in apoptotic processes, endodeoxyribonuclease, Tat protein, apolipoprotein, unfolded protein, ribosomal small subunit, core promoter binding, and DNA replication origin binding that are regulated by caspase 7 (*CASP7*), TatD DNase domain-containing 2 (*TATDN2*), nucleophosmin 1 (*NPM1*), heat shock protein family D member 1 (*HSPD1*), and RNA polymerase I transcription factor 3 (*RRN3*), were highly expressed in asthma patients after ICS/LABA treatment and normal subjects. The heatmap is shown in Fig. 1.

## Discussion

**Figure 2:**
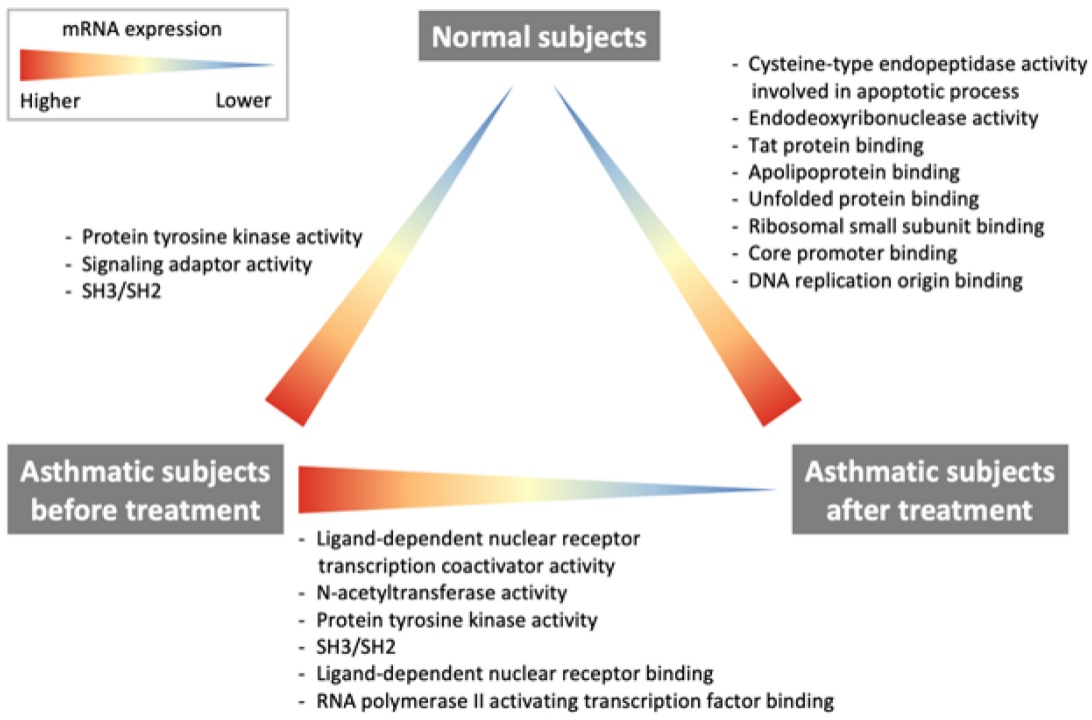
Differences in the RNA transcriptomes of asthmatic patients and healthy subjects.

The current study demonstrates some important findings regarding changes in RNA transcription, which are summarized in Fig. 2. The comparison of RNA transcriptome profiles of pre-treatment asthma patients and healthy subjects revealed that patients with asthma had higher protein tyrosine kinase activity. The comparison of post-treatment and baseline profiles of asthma patients showed that ligand-dependent nuclear receptor transcription coactivator activity, NAT activity, protein tyrosine kinase activity as well as ligand-dependent nuclear receptor, RNA polymerase II-activating transcription factor were downregulated after treatment with ICS/LABAs. However, the comparison between post-treatment asthma patients and healthy subjects showed that the cysteine-type endopeptidase activity involved in the apoptotic process, endodeoxyribonuclease activity, Tat protein, apolipoprotein, unfolded protein, ribosomal small subunit, core promoter binding, and DNA replication origin binding were still overexpressed in post-treatment asthma patients.

Protein tyrosine kinases play essential roles in the proliferation and activation of inflammatory and airway cells. Protein tyrosine kinase signaling is critical in allergic inflammation 11. The activation of receptor tyrosine kinases by ligand-induced dimerization and tyrosine phosphorylation leads to the recruitment and activation of signaling molecules containing SH2, SH3, and other domains 12. Tyrosine kinases are involved in responses to stimuli, and catalyze protein tyrosine phosphorylation to control intracellular signal transduction in response to many exogenous and endogenous stimuli 13. The stimulation of tyrosine kinases is the earliest response to immunoreceptor activation in eosinophils, mast cells, T cells, and B cells, which are important in the pathogenesis of asthma 11. The extent of tyrosine phosphorylation indicates the level of epithelial injury in asthma, and the levels of tyrosine kinases reflect the activation of stress signaling pathways 11. Considering airway inflammation, receptor tyrosine kinases are also important for airway remodeling 12. The activation of receptor tyrosine kinases is known to lead to hyperplasia of airway epithelial cells, smooth muscle cells, and goblet cells 12. In the current study, we demonstrated overexpression of protein tyrosine kinase in patients with asthma as compared with that in healthy subjects. This observation indicates that these patients had airway inflammation and remodeling. The expression of protein tyrosine kinase was downregulated after treatment with ICS/LABAs, indicating a decrease in mediators of airway inflammation and remodeling.

A transcription coactivator activates the transcription of specific gene sets by binding to a DNA-bound nuclear receptor. RNA polymerase II is a multiprotein complex that functions to transcribe the DNA into precursors of messenger RNA. Transcription factors are required to mediate its binding to upstream gene promoters of RNA polymerase II to initiate transcription. Corticosteroids work by binding to the intracellular glucocorticoid receptor (GR), followed by translocation to the nucleus and binding to glucocorticoid response elements to regulate the transcription of downstream genes 14, 15. This transactivation process decreases the induction of RNA polymerase II-mediated gene transcription and inhibits the expression of pro-inflammatory cytokines 14. In addition to ICS, LABAs can also induce activation of GR nuclear translocation 16. Therefore, the combination of ICS and LABA can augment the action of GR nuclear translocation in airway epithelial cells and macrophages 16. In the present study, the downregulation of nuclear receptor transcription coactivator and RNA polymerase II transcription factor activity in asthmatic patients after ICS-LABA treatment.

NATs are reported to play an important role in atopic diseases, including asthma 17. NATs are involved in the conversion of serotonin to melatonin and influence the formation of protein adducts, which mediates susceptibility to allergic inflammation 17. Increased acetylation was observed in contact-allergic patients, suggesting that acetylation may enhance sensitization 18. Over-representation of acetylators was found in patients with atopic disease 18. NATs were also found to be associated with extrinsic asthma 18. In the current study, the expression of NATs decreased in asthmatic patients after treatment with ICS/LABA. This is the first study to investigate this phenomenon in patients with asthma. This result indicates that allergy has improved after ICS-LABA treatment.

Cunningham et al. studied the importance of the enzymatic activity of cysteine proteases in asthma 19. These authors showed that cysteine protease activity initiates and drives allergic responses and that activation with L-cysteine leads to a significant increase in the induction of IgE and IgG, eosinophilia, interleukin (IL)-4, IL-5, and IL-10 in mouse bronchoalveolar lavage fluid after papain challenge 19. Therefore, cysteine protease activity is an adjuvant for Th2 responses 19. Th2 cells of the respiratory tract play important roles in mediating fulminant pulmonary allergic reactions 19. The Th2-based antigen presentation of papain by cysteine protease-activated basophils responds to the protease; this phenomenon can augment the response to allergenic proteins 19. House dust mites are considered one of the main allergens of active cysteine proteases 19. Exposure to house dust mites can cause an increase in cysteine proteases and trigger an allergic immune response 19. In the current study, asthmatic patients were stable after ICS-LABAs. However, they still showed higher cysteine-type endopeptidase activity. This indicates that the stimulation of house dust mites still exists. ICS-LABA treatment improved symptoms and airway inflammation but did not completely reduce the effects of allergens. Therefore, we recommend that the non-pharmacological management of allergen removal is still important for patients with asthma.

We compared post-treatment asthmatic patients with healthy subjects, and found that the expression of apolipoprotein was higher after treatment with ICS/LABA. Apolipoprotein plays a major role in lipid metabolism and exerts both pro- and anti-inflammatory effects 20. Apolipoprotein regulates inflammation, which may be dependent on cell types and disease models 20. Gordon et al. suggested that apolipoprotein functions as an endogenous danger signal that activates the inflammasome of macrophages to secrete IL-1β and induces airway inflammation in asthmatic subjects 21. Kalchiem-Dekel et al. also revealed that apolipoprotein mediates the inflammatory pathway of the Toll-like receptor 4-C-X-C motif chemokine ligand 5 axis in airway epithelial cells 22. However, Zhao et al. showed that apolipoprotein is a negative modulator of allergic airway inflammation in asthmatic mice 23, and apolipoprotein levels were lower in patients with atopic asthma than in healthy subjects 24. Thus, the role of apolipoprotein in asthma remains controversial. In our current study, we found that asthmatic subjects still had higher levels of apolipoprotein after ICS-LABA treatment. However, the clinical significance remains to be determined.

Endoplasmic reticulum (ER) stress and unfolded protein response (UPR) are also important in asthma 25. Many stimuli such as cigarette smoke, house dust mite, and fungal or viral pathogens may trigger ER stress and downstream signaling pathways 25. This phenomenon results in airway inflammation, remodeling, and hyperactivity in asthmatic patients 25. Therefore, ER stress with UPR activation promotes changes in airway allergic inflammatory and remodeling responses during allergic triggers 25. In our study, although the symptoms of these patients were controlled by ICS/LABAs, their UPR level was still higher than that of healthy subjects. All patients were non-smokers and had no active infections. We suggest that the higher UPR is attributed to the stimulation of environmental allergens. Therefore, the non-pharmacological management of allergen removal is important for patients with asthma.

In adults, asthma tends to be more severe in women than in men in terms of prevalence, severity, and duration 26. Different physiology and behaviors such as smoking, occupation, lifestyle, and diet may result in these differences 26. Female sex hormones linked to poor asthma control 26. Estrogen and progesterone promote asthma pathogenesis and worsen asthmatic symptoms, while testosterone prevents the inflammatory process of asthma 26. Different gene expression may be another factor in different manifestations of asthma. Tyrosine kinases are involved in regulating inflammatory responses, which are important in asthma. Activation of tyrosine kinases is an early and essential event in immune cell immune receptor signaling 27. Sahar et al. performed mRNA analysis of asthma and revealed that g.25827G>A serine tyrosine kinase mutation were more prevalent in women than men 27. However, the clinical significance of this mutational difference is unclear. Hamilton et al. performed bronchial biopsies in 9 patients with mild asthma (2 men, 7 women) and 9 patients with moderate asthma (5 men, 4 women), and there was no difference in phosphotyrosine levels between men or women 13. No evidence of gender differences in the distribution of NAT alleles and genotypes in asthma 18. However, house dust mites are considered to be one of the main allergens of active cysteine proteases 19. Women spend more time than men doing housework and are therefore more exposed to household allergens such as house dust 26. The role of apolipoprotein expression in different gender of asthmatic patients is unknown. One study of apolipoprotein expression in mice lungs showed higher levels apolipoprotein expression in females mice 28. There are no studies on gender-specific ER stress in asthma. However, in diabetes, women show higher expression of genes involved in protein synthesis and folding than men 29. Dysfunction of the protein folding machinery triggers ER stress, and there may be gender differences in cellular ER stress responses 29. In the current study, we randomly recruited newly diagnosed asthmatic patients, but more women with asthma (7 patients) were included in the analysis than men (2 patients). Therefore, the mRNA expression of different genders after treatment in asthmatic patients should be considered. Further studies should be performed to compare mRNA expression across genders.

## Clinical implication

According to our study, asthma patients under standard control and a well-controlled status still had higher cysteine-type endopeptidase activity involved in the apoptotic process, endodeoxyribonuclease activity, Tat protein, apolipoprotein, unfolded protein, ribosomal small subunit, core promoter binding, and DNA replication origin binding than healthy subjects. This result indicates that symptom control does not mean that inflammation has been completely controlled. Therefore, the development of an inflammatory marker strategy may be helpful. Heaney et al. performed a biomarker strategy (T2 biomarkers of fractional exhaled nitric oxide, blood eosinophils, and serum periostin) to adjust the corticosteroid dose and compared it with a symptom-based algorithm 30. However, these authors suggested that T2 biomarker-based corticosteroid adjustment may not reduce the corticosteroid dose in many patients 30. According to our study, higher gene expression is not limited to T2 biomarkers but also includes pathways related to airway remodeling or ER stress in asthma patients. In our study, RNA transcriptome analysis might help clinicians understand the comprehensive inflammation or airway remodeling status of asthma patients. However, the cost of RNA transcriptome analysis is too high to be widely used in the clinical management of these patients. Even so, this research opens up related research on these issues in patients with asthma.

## Limitations of the study

This study has several limitations. First, the sample size was too small, which may have caused statistical bias. The descriptive statistics had showed difference between groups but had no statistical significance. We attribute these phenomena due to the variance between the groups may quite different. To obtain accurate RNA transcriptome results, we had strict patient inclusion and exclusion criteria. In addition, given the high cost of RNA transcriptome analysis, it is difficult to perform large-scale testing on patients. Second, RNA transcriptome analysis generates a lot of data, but its clinical significance still needs to be further explored. Through this research, we identified several important mediators of airway inflammation and remodeling. Studies on these mediators are limited. We have paved a way for future relevant animal or clinical studies. Third, most of the enrolled patients were women. There may be differences in mRNA expression between genders. Further studies should be performed to compare mRNA expression between genders.

## Conclusions

The activity of protein tyrosine kinase was higher in asthmatic patients and downregulated by ICS-LABAs. Treatment with ICS-LABAs also decreased nuclear receptor transcription coactivator and NAT activities. Although the symptoms of these asthmatic patients were under control, they still had higher cysteine-type endopeptidase activity and unfolded protein. Thus, these patients were still under stimulation by environmental allergens. The combination of non-pharmacological management and allergen removal is, therefore, a complete strategy.

## Figures and Tables

**Table 1:** Clinical and demographic characteristics of patients

	Asthmatic patients	Healthy subjects	*p*-valve
**Sex**			0.491
Man, n (%)	2 (22.2%)	1 (50%)	
Woman, n (%)	7 (77.8%)	1 (50%)	
Age, yr.	44.9 (± 14.1)	38.5 (±4.9)	0.3203
Body height (cm)	157.8 (±7.5)	166.8 (±6.0)	0.2273
Body weight (kg)	61.6 (±13.3)	70.8 (±19.4)	0.625
BMI	24.6 (± 4.7)	25.2 (±5.2)	0.9034
**Allergen, n (%)**			
Dust mite	4.3 (±6.4)	0.24(±2.5)	0.0923
Cat dander	15.0 (±35.4)	0.0 (±0.0)	0.2702
Dog dander	4.9 (±13.5)	0.01(±0.01)	0.3406
Cockroach	0.4 (±0.7)	0.09(±0.04)	0.1802
Fungus	0.1 (±0.1)	0.02(±0.01)	0.3032
Bermuda grass	0.05(±0.08)	0.02(±0.01)	0.3721
**Pre-bronchodilator**			
FVC (L)	3.1 (±0.6)	3.7 (±1.4)	0.6072
FVC (%)	95.6 (±12.9)	92.5 (±13.4)	0.8539
FEV1 (L)	2.4 (±0.5)	3.1 (±1.0)	0.3965
FEV1 (%)	87.7 (±14.3)	93.0 (±7.1)	0.1836
FVC/FEV1	77.1( ±9.1)	85.0 (±5.7)	0.2952
**Post-bronchodilator**			
FVC (L)	3.0 (±0.7)	3.7 (±1.3)	0.4974
FVC (%)	90.8 (±13.0)	92.0 (±9.9)	0.2751
FEV1 (L)	2.4 (±0.5)	3.2 (±1.0)	0.3706
FEV1 (%)	83.1 (±10.3)	97.5 (±6.4)	0.1027
FVC/FEV1	77.3 (±9.7)	88.5 (±4.9)	0.0967

Data are presented as mean± SD or n (%).Chi-square used for categorical data comparison. Welch's t-test used for independent groups' comparison).

**Table 2:** Differences among healthy subjects, pre-treatment and post-treatment asthmatic patients

Lab data	Healthy subjects	Asthma, pre-treatment	Asthma, post-treatment	*p*-value*	*p*-value **	*p*-value***
WBC (10^3^/uL)	5415.0 (±205.1)	5457.8 (±1977.3)	6837.8 (±1962.1)	0.9509	0.0639	0.0039
Hemoglobin(10^6^/uL)	14.0 (±2.0)	13.1 (±1.6)	13.2 (±1.6)	0.6315	0.6777	0.3828
Platelet (10^3^/uL)	217.0 (±21.2)	279.1 (±54.4)	293.1 (±70.6)	0.0465	0.03023	0.3008
Basophil (%)	1.1 (±0.3)	0.7 (±0.4)	0.6 (±0.2)	0.2744	0.1829	0.0937
Neutrophil (%)	58.2 (±7.5)	51.2 (±8.4)	56.9 (±6.6)	0.3862	0.8474	0.0273
Eosinophil (%)	2.0 (±0.4)	4.7 (±2.9)	3.9 (±2.8)	0.0209	0.0802	0.1641
Lymphocyte (%)	31.3 (±9.5)	37.1 (±7.3)	33.3 (±6.4)	0.5399	0.8129	0.1641
IgE (IU/mL)	31.5 (±31.4)	426.6 (±658.0)	409.1(±622.0)	0.1101	0.1069	0.5703
Na (mEq/L)	135.5 (±6.4)	139.4 (±1.8)	139.0 (±1.4)	0.5462	0.5786	0.3125
K (mEq/L)	3.8 (±0.1)	4.1 (±0.3)	4.1 (±0.3)	0.0931	0.1407	0.625
CRP (mg/L)	0.1 (±0.0)	0.2 (±0.1)	0.3 (±0.4)	0.2629	0.2999	0.5625
ACT	25.0(±0.0)	16.2(±2.9)	20.1(±2.5)	<0.001	0.0003	0.0156

Data are presented as mean± SD or n (%). Welch's T Test (for independent groups' comparison) and Wilcoxon signed rank T Test (for paired data companion). Definition of abbreviations: PEFR: peak expiratory flow ratio; ACT: asthma control test. *p*-valve^*^ Pretreatment asthmatic vs. healthy subjects; *p* -valve^ **^ Post-treatment asthmatic vs. healthy subjects;* p* -valve^***^Pretreatment vs. Post-treatment asthmatic patients.

**Table 3:** Difference of RNA transcription between pre-treatment asthmatic vs healthy subjects

GO. Term	Gene	Description	Category	Post-FC	p-value
GO:0004713	SLA2	protein tyrosine kinase activity	UP	1.88	0.052
GO:0004715	SLA2	non-membrane spanning protein tyrosine kinase activity	UP	1.88	0.015
GO:0060090	SLA2	binding, bridging	UP	1.88	0.052
GO:0030674	SLA2	protein binding, bridging	UP	1.88	0.047
GO:0035591	SLA2	signaling adaptor activity	UP	1.88	0.023
GO:0005070	SLA2	SH3/SH2 adaptor activity	UP	1.88	0.017

**Abbreviations:** Fold changes: FC: fold changes; UP: upper expression**;** SRC homology 3: SH3; SRC homology 2 SH2; Src Like Adaptor 2: SLA2.

**Table 4:** Changes of RNA transcription in asthma subjects before and after ICS/LABA treatment

GO. Term	Gene	Description	Category	Post-FC	p-value
GO:0030374	TADA3	ligand-dependent nuclear receptor transcription coactivator activity	DOWN	0.90	0.018
GO:0016746	TADA3	transferase activity, transferring acyl groups	DOWN	0.90	0.088
GO:0016747	TADA3	transferase activity, transferring acyl groups other than amino-acyl groups	DOWN	0.90	0.074
GO:0016407	TADA3	acetyltransferase activity	DOWN	0.90	0.035
GO:0016410	TADA3	N-acyltransferase activity	DOWN	0.90	0.035
GO:0008080	TADA3	N-acetyltransferase activity	DOWN	0.90	0.029
GO:0034212	TADA3	peptide N-acetyltransferase activity	DOWN	0.90	0.022
GO:0061733	TADA3	peptide-lysine-N-acetyltransferase activity	DOWN	0.90	0.020
GO:0004402	TADA3	histone acetyltransferase activity	DOWN	0.90	0.019
GO:0016922	TADA3	ligand-dependent nuclear receptor binding	DOWN	0.90	0.007
GO:0004713	SLA2	protein tyrosine kinase activity	DOWN	0.78	0.062
GO:0004715	SLA2	non-membrane spanning protein tyrosine kinase activity	DOWN	0.78	0.018
GO:0060090	SLA2	binding, bridging	DOWN	0.78	0.062
GO:0030674	SLA2	protein binding, bridging	DOWN	0.78	0.056
GO:0035591	SLA2	signaling adaptor activity	DOWN	0.78	0.028
GO:0005070	SLA2	SH3/SH2 adaptor activity	DOWN	0.78	0.020
GO:0005521	IFI27	lamin binding	DOWN	0.08	0.005
GO:0001085	IFI27	RNA polymerase II transcription factor binding	DOWN	0.08	0.040
GO:0033613	IFI27	activating transcription factor binding	DOWN	0.08	0.022
GO:0001102	IFI27	RNA polymerase II activating transcription factor binding	DOWN	0.08	0.014

**Abbreviations:** Fold changes: FC; DOWN: down expression; SRC homology 3: SH3; SRC homology 2 SH2; Src Like Adaptor 2: SLA2; Transcriptional Adaptor 3: TADA3; Interferon Alpha Inducible Protein 27: IFI27.

**Table 5:** Differences of RNA transcription in post-treatment asthmatic subjects vs healthy controllers

GO. Term	Gene	Description	Category	Post-FC	p-value
GO:0097153	CASP7	cysteine-type endopeptidase activity involved in apoptotic process	UP	1.49	0.011
GO:0097200	CASP7	cysteine-type endopeptidase activity involved in execution phase of apoptosis	UP	1.49	0.009
GO:0016888	TATDN2	endodeoxyribonuclease activity, producing 5'-phosphomonoesters	UP	1.25	0.008
GO:0030957	NPM1	Tat protein binding	UP	1.30	0.008
GO:0043024	NPM1	ribosomal small subunit binding	UP	1.31	0.001
GO:0001047	NPM1//RRN3	core promoter binding	UP	1.30	0.007
GO:0034185	HSPD1	apolipoprotein binding	UP	1.31	0.012
GO:0051787	HSPD1	misfolded protein binding	UP	1.30	0.010
GO:0003688	HSPD1	DNA replication origin binding	UP	1.30	0.001
GO:0051082	NPM1//HSPD1	unfolded protein binding	UP	1.31	0.003

**Abbreviations:** Fold changes: FC; UP: up expression; Caspase 7 (CASP7), TatD DNase Domain Containing 2 (TATDN2), Nucleophosmin 1 (NPM1), (Heat Shock Protein Family D Member 1 (HSPD1), and RNA polymerase I transcription factor 3 (RRN3).

## References

[1] Enilari O, Sinha S (2019). The Global Impact of Asthma in Adult Populations. *Ann Glob Health*.

[2] Kim SH, Lee T, Jang AS, Park CS, Jung JW, Kim MH, Kwon JW, Moon JY, Yang MS, Lee J (2021). Pragmatic Randomized Controlled Trial for Stepping Down Asthma Controller Treatment in Patients Controlled with Low-Dose Inhaled Corticosteroid and Long-Acting β(2)-Agonist: Step-Down of Intervention and Grade in Moderate Asthma Study. *The journal of allergy and clinical immunology In practice*.

[3] Henderson I, Caiazzo E, McSharry C, Guzik TJ, Maffia P (2020). Why do some asthma patients respond poorly to glucocorticoid therapy?. *Pharmacological research*.

[4] Hou L, Hao H, Huang G, Liu J, Yu L, Zhu L, Shen H, Zhang M (2021). The value of small airway function parameters and fractional exhaled nitric oxide for predicting positive methacholine challenge test in asthmatics of different ages with FEV(1) ≥ 80% predicted. *Clin Transl Allergy*.

[5] Ye L, Gao X, Tu C, Du C, Gu W, Hang J, Zhao L, Jie Z, Li H, Lu Y (2021). Comparative analysis of effectiveness of asthma control test-guided treatment versus usual care in patients with asthma from China. *Respir Med*.

[6] Bolger AM, Lohse M, Usadel B (2014). Trimmomatic: a flexible trimmer for Illumina sequence data. *Bioinformatics*.

[7] Langmead B, Salzberg SL (2012). Fast gapped-read alignment with Bowtie 2. *Nat Methods*.

[8] Li B, Dewey CN (2011). RSEM: accurate transcript quantification from RNA-Seq data with or without a reference genome. *BMC Bioinformatics*.

[9] Leng N, Dawson JA, Thomson JA, Ruotti V, Rissman AI, Smits BM, Haag JD, Gould MN, Stewart RM, Kendziorski C (2013). EBSeq: an empirical Bayes hierarchical model for inference in RNA-seq experiments. *Bioinformatics*.

[10] Yu G, Wang LG, Han Y, He QY (2012). clusterProfiler: an R package for comparing biological themes among gene clusters. *Omics*.

[11] Duan W, Kuo IC, Selvarajan S, Chua KY, Bay BH, Wong WS (2003). Antiinflammatory effects of genistein, a tyrosine kinase inhibitor, on a guinea pig model of asthma. *Am J Respir Crit Care Med*.

[12] Wong WS, Leong KP (2004). Tyrosine kinase inhibitors: a new approach for asthma. *Biochim Biophys Acta*.

[13] Hamilton LM, Puddicombe SM, Dearman RJ, Kimber I, Sandström T, Wallin A, Howarth PH, Holgate ST, Wilson SJ, Davies DE (2005). Altered protein tyrosine phosphorylation in asthmatic bronchial epithelium. *The European respiratory journal*.

[14] Cazzola M, Rogliani P, Calzetta L, Matera MG (2020). Pharmacogenomic Response of Inhaled Corticosteroids for the Treatment of Asthma: Considerations for Therapy. *Pharmgenomics Pers Med*.

[15] Wadhwa R, Dua K, Adcock IM, Horvat JC, Kim RY, Hansbro PM (2019). Cellular mechanisms underlying steroid-resistant asthma. *Eur Respir Rev*.

[16] Usmani OS, Ito K, Maneechotesuwan K, Ito M, Johnson M, Barnes PJ, Adcock IM (2005). Glucocorticoid receptor nuclear translocation in airway cells after inhaled combination therapy. *Am J Respir Crit Care Med*.

[17] Batra J, Ghosh B (2008). N-acetyltransferases as markers for asthma and allergic/atopic disorders. *Curr Drug Metab*.

[18] Nacak M, Aynacioglu AS, Filiz A, Cascorbi I, Erdal ME, Yilmaz N, Ekinci E, Roots I (2002). Association between the N-acetylation genetic polymorphism and bronchial asthma. *British journal of clinical pharmacology*.

[19] Cunningham PT, Elliot CE, Lenzo JC, Jarnicki AG, Larcombe AN, Zosky GR, Holt PG, Thomas WR (2012). Sensitizing and Th2 adjuvant activity of cysteine protease allergens. *Int Arch Allergy Immunol*.

[20] Qin S, Pu Q, Wang Z, Wu M (2020). Apolipoprotein E in Asthmatic Inflammatory Response: Friend or Foe?. *American journal of respiratory cell and molecular biology*.

[21] Gordon EM, Yao X, Xu H, Karkowsky W, Kaler M, Kalchiem-Dekel O, Barochia AV, Gao M, Keeran KJ, Jeffries KR (2019). Apolipoprotein E is a concentration-dependent pulmonary danger signal that activates the NLRP3 inflammasome and IL-1β secretion by bronchoalveolar fluid macrophages from asthmatic subjects. *J Allergy Clin Immunol*.

[22] Kalchiem-Dekel O, Yao X, Barochia AV, Kaler M, Figueroa DM, Karkowsky WB, Gordon EM, Gao M, Fergusson MM, Qu X (2020). Apolipoprotein E Signals via TLR4 to Induce CXCL5 Secretion by Asthmatic Airway Epithelial Cells. *American journal of respiratory cell and molecular biology*.

[23] Zhao CC, Xu J, Xie QM, Fan XY, Fei GH, Wu HM (2020). Apolipoprotein E negatively regulates murine allergic airway inflammation via suppressing the activation of NLRP3 inflammasome and oxidative stress. *Int Immunopharmacol*.

[24] Bhowmik M, Majumdar S, Dasgupta A, Gupta Bhattacharya S, Saha S (2019). Pilot-Scale Study Of Human Plasma Proteomics Identifies ApoE And IL33 As Markers In Atopic Asthma. *J Asthma Allergy*.

[25] Bradley KL, Stokes CA, Marciniak SJ, Parker LC, Condliffe AM (2021). Role of unfolded proteins in lung disease. *Thorax*.

[26] Jenkins CR, Boulet LP, Lavoie KL, Raherison-Semjen C, Singh D (2022). Personalized Treatment of Asthma: The Importance of Sex and Gender Differences. *The journal of allergy and clinical immunology In practice*.

[27] Sahar N, Bibi S, Masood N, Faryal R (2019). Status of serine tyrosine kinase at germline and expressional levels in asthma patients. *Molecular biology research communications*.

[28] Yao X, Gordon EM, Figueroa DM, Barochia AV, Levine SJ (2016). Emerging Roles of Apolipoprotein E and Apolipoprotein A-I in the Pathogenesis and Treatment of Lung Disease. *American journal of respiratory cell and molecular biology*.

[29] Brownrigg G JJ, Rideout E (2021). Sex differences in β-cells endoplasmic reticulum stress response. *CJD*.

[30] Heaney LG, Busby J, Hanratty CE, Djukanovic R, Woodcock A, Walker SM, Hardman TC, Arron JR, Choy DF, Bradding P (2021). Composite type-2 biomarker strategy versus a symptom-risk-based algorithm to adjust corticosteroid dose in patients with severe asthma: a multicentre, single-blind, parallel group, randomised controlled trial. *Lancet Respir Med*.

